# Longitudinal changes in sodium concentration and in clinical outcome in mild traumatic brain injury

**DOI:** 10.1093/braincomms/fcae229

**Published:** 2024-07-11

**Authors:** Teresa Gerhalter, Anna M Chen, Seena Dehkharghani, Rosemary Peralta, Mia Gajdosik, Alejandro Zarate, Tamara Bushnik, Jonathan M Silver, Brian S Im, Stephen P Wall, Guillaume Madelin, Ivan I Kirov

**Affiliations:** Bernard and Irene Schwartz Center for Biomedical Imaging, Department of Radiology, New York University Grossman School of Medicine, New York, NY 10016, USA; Center for Advanced Imaging Innovation and Research (CAI^2^R), Department of Radiology, New York University Grossman School of Medicine, New York, NY 10016, USA; Bernard and Irene Schwartz Center for Biomedical Imaging, Department of Radiology, New York University Grossman School of Medicine, New York, NY 10016, USA; Center for Advanced Imaging Innovation and Research (CAI^2^R), Department of Radiology, New York University Grossman School of Medicine, New York, NY 10016, USA; Vilcek Institute of Graduate Biomedical Sciences, New York University Grossman School of Medicine, New York, NY 10016, USA; Bernard and Irene Schwartz Center for Biomedical Imaging, Department of Radiology, New York University Grossman School of Medicine, New York, NY 10016, USA; Center for Advanced Imaging Innovation and Research (CAI^2^R), Department of Radiology, New York University Grossman School of Medicine, New York, NY 10016, USA; Department of Neurology, New York University Grossman School of Medicine, New York, NY 10016, USA; Bernard and Irene Schwartz Center for Biomedical Imaging, Department of Radiology, New York University Grossman School of Medicine, New York, NY 10016, USA; Center for Advanced Imaging Innovation and Research (CAI^2^R), Department of Radiology, New York University Grossman School of Medicine, New York, NY 10016, USA; Bernard and Irene Schwartz Center for Biomedical Imaging, Department of Radiology, New York University Grossman School of Medicine, New York, NY 10016, USA; Center for Advanced Imaging Innovation and Research (CAI^2^R), Department of Radiology, New York University Grossman School of Medicine, New York, NY 10016, USA; Department of Rehabilitation Medicine, New York University Grossman School of Medicine, New York, NY 10016, USA; Department of Rehabilitation Medicine, New York University Grossman School of Medicine, New York, NY 10016, USA; Department of Psychiatry, New York University Grossman School of Medicine, New York, NY 10016, USA; Department of Rehabilitation Medicine, New York University Grossman School of Medicine, New York, NY 10016, USA; Ronald O. Perelman Department of Emergency Medicine, New York University Grossman School of Medicine, New York, NY 10016, USA; Bernard and Irene Schwartz Center for Biomedical Imaging, Department of Radiology, New York University Grossman School of Medicine, New York, NY 10016, USA; Center for Advanced Imaging Innovation and Research (CAI^2^R), Department of Radiology, New York University Grossman School of Medicine, New York, NY 10016, USA; Vilcek Institute of Graduate Biomedical Sciences, New York University Grossman School of Medicine, New York, NY 10016, USA; Bernard and Irene Schwartz Center for Biomedical Imaging, Department of Radiology, New York University Grossman School of Medicine, New York, NY 10016, USA; Center for Advanced Imaging Innovation and Research (CAI^2^R), Department of Radiology, New York University Grossman School of Medicine, New York, NY 10016, USA; Vilcek Institute of Graduate Biomedical Sciences, New York University Grossman School of Medicine, New York, NY 10016, USA; Department of Neurology, New York University Grossman School of Medicine, New York, NY 10016, USA

**Keywords:** quantitative ^23^Na MRI, total sodium concentration, longitudinal clinical outcome assessment, cognitive test, post-concussion symptoms

## Abstract

Ionic imbalances and sodium channel dysfunction, well-known sequelae of traumatic brain injury (TBI), promote functional impairment in affected subjects. Therefore, non-invasive measurement of sodium concentrations using ^23^Na MRI has the potential to detect clinically relevant injury and predict persistent symptoms. Recently, we reported diffusely lower apparent total sodium concentrations (aTSC) in mild TBI patients compared to controls, as well as correlations between lower aTSC and worse clinical outcomes. The main goal of this study was to determine whether these aTSC findings, and their changes over time, predict outcomes at 3- and 12-month from injury. Twenty-seven patients previously studied with ^23^Na MRI and outcome measures at 22 ± 10 days (average ± standard deviation) after injury (visit-1, v1) were contacted at 3- (visit-2, v2) and 12-month after injury (visit-3, v3) to complete the Rivermead post-concussion symptoms questionnaire (RPQ), the extended Glasgow outcome scale (GOSE), and the brief test of adult cognition by telephone (BTACT). Follow-up ^1^H and ^23^Na MRI were additionally scheduled at v2. Linear regression was used to calculate aTSC in global grey and white matters. Six hypotheses were tested in relation to the serial changes in outcome measures and in aTSC, and in relation to the cross-sectional and serial relationships between aTSC and outcome. Twenty patients contributed data at v2 and fifteen at v3. Total RPQ and composite BTACT *z*-scores differed significantly for v2 and v3 in comparison to v1 (each *P* < 0.01), reflecting longitudinally reduced symptomatology and improved performance on cognitive testing. No associations between aTSC and outcome were observed at v2. Previously lower grey and white matter aTSC normalized at v2 in comparison to controls, in line with a statistically detectable longitudinal increase in grey matter aTSC between v1 and v2 (*P* = 0.0004). aTSC values at v1 predicted a subset of future BTACT subtest scores, but not future RPQ scores nor GOSE-defined recovery status. Similarly, aTSC rates of change correlated with BTACT rates of change, but not with those of RPQ. Tissue aTSC, previously shown to be diffusely decreased compared to controls at v1, was no longer reduced by v2, suggesting normalization of the sodium ionic equilibrium. These changes were accompanied by marked improvement in outcome. The results support the notion that early aTSC from ^23^Na MRI predicts future BTACT, but not RPQ scores, nor future GOSE status.

## Teaser

Gerhalter *et al*. reported that mild traumatic brain injury (mTBI) patients, initially scanned within weeks of injury, exhibit a significant normalization of apparent total sodium concentration after 3 months. Notably, these sodium changes predict outcome performance on brief test of adult cognition by telephone (BTACT), emphasizing the study’s clinical relevance.

## Graphical Abstract

**Graphical Abstract:**
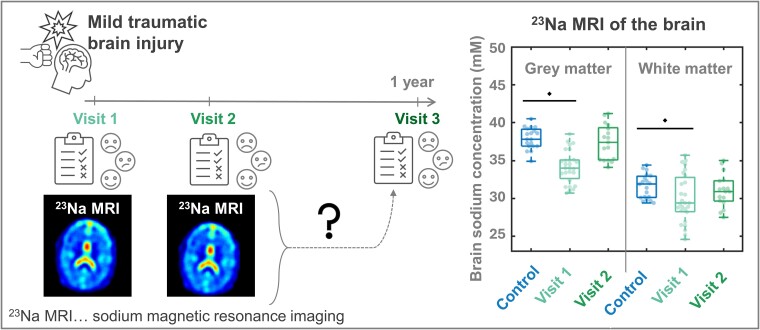

## Introduction

It is estimated that half of the global population will suffer at least one traumatic brain injury (TBI) over their lifetime.^1^ Most cases will be classified as mild traumatic brain injury (mTBI),^1^ the least severe subtype which, despite its designation, includes a sub-population of patients at high, but poorly understood risk of ongoing post-concussive symptoms (PCS) persisting beyond the usual recovery period of several weeks.^2,3^ A variety of domains (e.g. somatic, cognitive and psychological) may be affected,^4^ negatively impacting global functioning and quality of life. Thus, there remains a need for non-invasive biomarkers to forecast symptom persistence and clinical outcomes in individual patients, as well as to guide treatment and project therapeutic response.

Unfortunately, years of efforts to improve the clinical utility of MRI in mTBI have not resulted in a recommendation for its routine use in prognostication.^5^ Conventional MRI exams are typically normal or unrevealing, even among those with long-term PCS,^6^ and while image acquisitions sensitive to iron deposits such as susceptibility-weighted imaging (SWI) may improve sensitivity, findings do not correlate strongly with outcome.^7,8^ Quantitative magnetic resonance (MR) methods, such as diffusion tensor imaging and MR spectroscopy, have contributed to the understanding of injury mechanisms, but have not gained widespread adoption, motivating exploration into alternative imaging techniques.

We recently investigated whether known changes in ionic homeostasis after mTBI could be detected *in vivo* with sodium (^23^Na) MRI, and whether any such changes had the potential to serve as a surrogate marker of clinical outcome.^9^ The study was based on the knowledge that Na^+^ channels and ionic homeostasis are affected after TBI,^10,11^ and that such changes may be tied to clinical deficits.^12-14^ In our cross-sectional, prospective case-control study we found lower apparent total sodium concentration (aTSC) in patients compared to controls, throughout the global grey matter (GM) and white matter (WM). Additionally, lower aTSC correlated with worse scores on neuropsychological assessment and differentiated non-recovered patients from controls. These results lent support to the potential use of ^23^Na MRI as an imaging biomarker in the setting of TBI.^9^

Validation of candidate biomarkers demands greater understanding of their evolution over time, including validation of their potential to predict intermediate and longer-term outcomes. In this study, we assess these characteristics of global GM and WM aTSC during longitudinal subject clinical and imaging encounters, including two ^23^Na MRI visits: Approximately 1 month after injury (visit-1, v1, as reported previously^9^), and at 3 months following injury (visit-2, v2); and three clinical encounters to collect outcome measures of global functioning, symptomatology and neuropsychological testing [v1, v2, and at 1 year after injury (visit-3, v3)]. We tested six *a priori* hypotheses established in our prospective funded study (H1–H6, Table 1) related to the serial changes in outcome measures and in aTSC (H1–H3); and related to the cross-sectional and serial relationships between aTSC and outcome (H4–H6). Our overall goal was to assess the utility of global aTSC for the prediction of intermediate- and long-term outcomes following mTBI.

**Table 1:** Summary of study hypotheses with their corresponding variables, statistical tests and results

	Investigated parameter	Hypothesis	Statistical test	Results
H1: patient outcome changes over time	v2−v1 for BTACT and RPQ v3−v1 for BTACT and RPQ	Improvement in all outcome measures over time, consistent with the literature	WSRT	Supported
H2: aTSC changes over time	v2−v1 for aTSC	Recovery of aTSC over time in the whole TBI group as outcome measures are also expected to improve, consistent with H1 and with a hypothesized biomarker property of aTSC	WSRT	Partially supported
H3: aTSC at v2	v2 for aTSC	Smaller effect sizes for the differences in aTSC between patients and controls, compared to the prior report, due to expectation of concurrent recovery of aTSC and of patient outcome scores	MW	Supported
H4: correlation of aTSC at v2 with outcome measures at v2	v2 aTSC versus v2 BTACT and RPQ	Lower aTSC in patients with symptoms or worse cognitive performance compared to matched controls	Spearman rank	Rejected
	v2 aTSC based on v2 GOSE (recovered or not)	Not recovered patients at v2 have lower aTSC at v2 compared to controls	MW	Rejected
H5: correlation of aTSC at v1 with outcome measures at v2 and v3	v1 aTSC versus v2 and v3 BTACT and RPQ	Lower aTSC at v1 correlates with worse clinical outcome at v2 and v3	Spearman	Partially supported
	v1 aTSC based on v2 GOSE (recovered or not)	Not recovered patients at v2 have lower aTSC at v1 compared to controls	MW	Supported
H6: correlation of aTSC changes with changes in outcome measures	v2−v1 aTSC versus v2−v1 BTACT and RPQ	Changes in aTSC in patients correlate with changes in clinical outcome measures	Spearman	Partially supported
	v2−v1 aTSC based on v2 GOSE (recovered or not)	Patients who did not recovered at v1, but recovered at v2, show a statistically different aTSC rate of change from those who failed to recover at v2	MW	Rejected

The different hypotheses were numbered (e.g., H1, H2…) to structure the results as outlined in the text. aTSC refers to both grey and white matter aTSC.

## Materials and methods

### Study population

**Figure 1:**
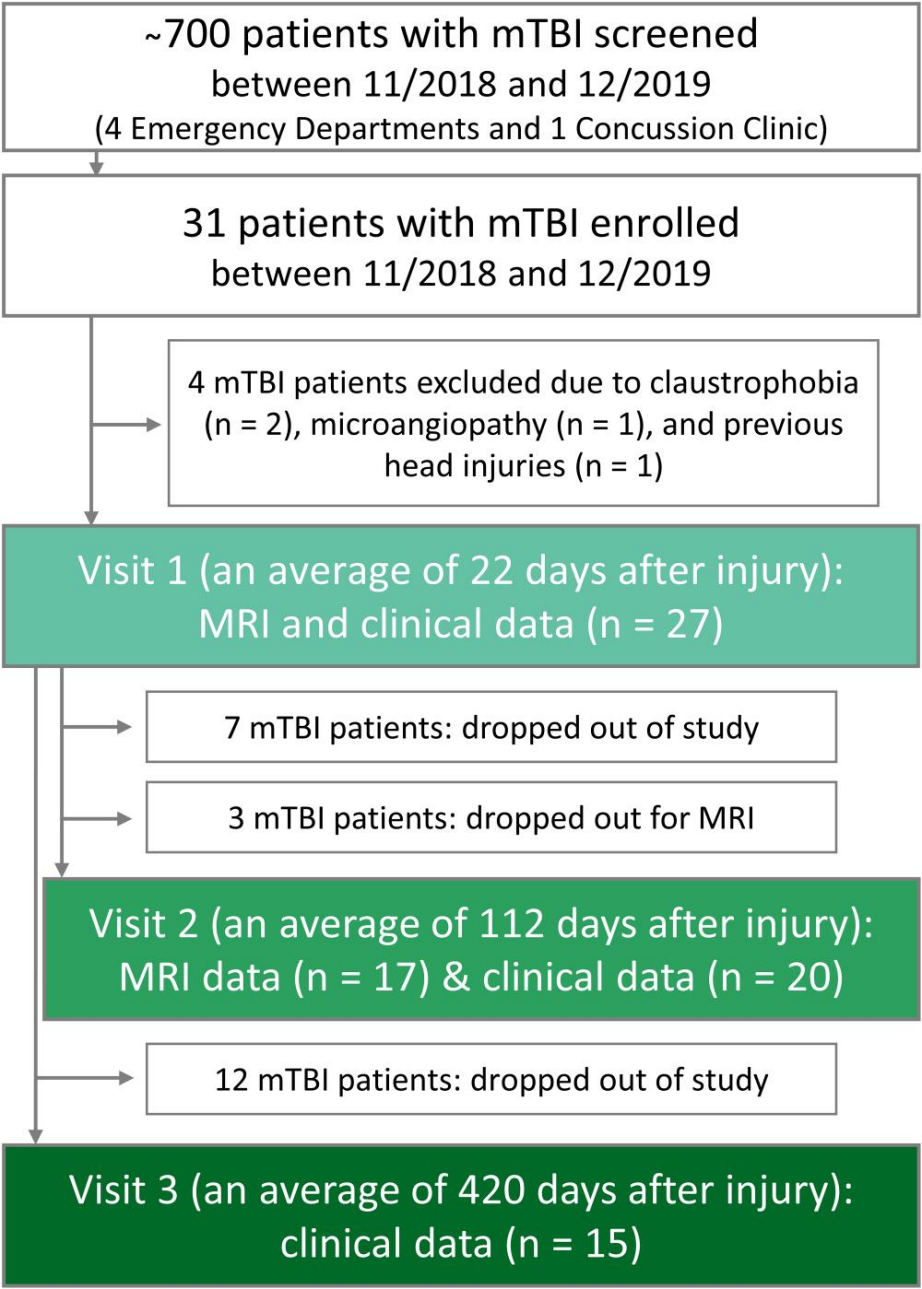
**Flow diagram of study design, enrolment and drop-out rates.** Patient recruitment took place from four metropolitan emergency departments and a concussion clinic. Eligible and willing patients underwent clinical outcome assessments at visits 1, 2 and 3; and MRI scanning at visits 1 and 2.

The Institutional Review Board approved this study and written informed consent was obtained from all participants. Recruitment details are described in our prior publication,^9^ and the number of screened and enrolled patients, as well as the basic study design, are provided in Fig. 1. Briefly, 31 patients and 19 matched controls were prospectively enrolled between November 2018 and December 2019 following the inclusion and exclusion criteria listed in Supplemental Material. For the current study, the enrolled patients were asked to return for two repeat visits, at 3 months (v2) and 1 year (v3) post-TBI.

### Clinical outcome assessments

The abbreviated injury scale (AIS) score was collected at the time of initial diagnosis by medical caretakers upon admission to the Emergency Department to describe the severity of the injury at presentation. At each of the three visits, outcome status was further assessed by trained study members (RP, AZ, MG) with the (i) 16-item Rivermead post-concussion symptoms questionnaire (RPQ),^15^ (ii) 8-point Glasgow outcome scale-extended (GOSE),^16^ and (iii) brief test of adult cognition by telephone (BTACT).^17,18^

The RPQ is used to describe the presence and severity of PCS [range: 0 (no symptoms)–64 (worst)]. We used the total RPQ score, as well as the scores from a three-factor structure as suggested by Smith–Seemiller and colleagues,^19^ dividing PCS into *cognitive* (forgetfulness, poor concentration, taking longer to think), *somatic* (headaches, double or blurred vision, sensitivity to noise, dizziness, nausea, sleep disturbance, fatigue) and *emotional* (irritability, depression, frustration, restlessness) domains. Symptoms must have first occurred after the injury or must have worsened after the injury (i.e. RPQ score > 1) to qualify as PCS.

The GOSE^16,20^ is used as a fundamental metric for gauging overall functional outcome following TBI and is an integral component of the National Institute of Neurological Disorders and Stroke (NINDS) common data elements.^21,22^ Comprising eight points, the GOSE evaluates the broader impact of TBI on factors such as independence, occupational capacity, social interactions and cognitive function. A score of eight signifies complete recovery and the resumption of a normal lifestyle, while descending scores from seven to three indicate escalating levels of mental and/or physical impairment. For our analysis, patients were categorized into ‘recovered’ (GOSE = 8) and ‘non-recovered’ (GOSE ≤ 7) groups, a common practice,^20,23^ also endorsed in a validation study^24^ utilizing data from the multicentre transforming research and clinical knowledge in traumatic brain injury consortium.

The BTACT is a cognitive testing battery comprised of six subtests, which provide measures of episodic and working memory, executive function, reasoning and processing speed.^18^ A standardized *z*-score was calculated for each subtest and then compiled into a BTACT composite *z*-score based on the population means and standard deviations from the MIDUS II cognitive study to account for age, education and sex. The BTACT has previously been used in mTBI research,^25^ and is part of the testing battery of the National Institute on Disability, Independent Living and Rehabilitation Research (NIDILRR)-funded TBI Model System centres.^26^

Assessments were performed face-to-face prior to the MRI exam unless institutional or other restraints prevented patients from completing the full battery in-person, in which case they were administered over the phone in the days following the in-person visit. Both the RPQ and GOSE can be conducted either in-person or by phone.^3^ Similarly, the mode of BTACT administration (phone or in-person) was left flexible due to its validity with either approach.^17^

### MRI data acquisition

As previously detailed,^9^ all participants were scanned on a 3 T scanner (MAGNETOM Prisma, Siemens Healthcare, Erlangen, Germany) with a 20-channel head coil (Siemens Healthcare, Erlangen, Germany) and a custom-made ^1^H/^23^Na dual-tuned birdcage head coil. Macroscopic injury was assessed with 2D-fluid attenuation inversion recovery (FLAIR: repetition/echo/inversion time = 9000/81/2500 ms, 30 slices, in-plane field-of-view = 220 × 220 mm^2^, voxel size = 0.7 × 0.7 × 5.0 mm³, acquisition time = 2:44 min); and susceptibility-weighted imaging (SWI: repetition/echo time = 28/20 ms, in-plane field-of-view = 220 × 220 mm^2^, voxel size = 0.7 × 0.7 × 3.0 mm³, acquisition time = 3:46 min). A 3D-T_1_-weighted magnetization prepared rapid gradient echo (MPRAGE: repetition/echo/inversion time = 2400/2.24/1060 ms, 208 slices, in-plane field-of-view = 256 × 256 mm^2^, voxel size = 0.8 mm isotropic, acquisition time = 6:38 min) was performed for co-registration with the ^23^Na images and cerebrospinal fluid (CSF), GM and WM tissue segmentation. For ^23^Na MRI, a global flip angle calibration^27^ and a manual B_0_ shim using the manufacturer’s ^1^H MRI-based B_0_-shimming routine were first performed. ^23^Na images were then acquired using non-Cartesian FLORET^28^ with ultra-short echo time: repetition/echo time = 100/0.2 ms, three hubs at 45°, number of interleaves/hubs = 26, nominal resolution = 6 mm isotropic, 46 averages, acquisition time = 5:59 min. For co-registration with the proton data, low-resolution ^1^H MPRAGE images were acquired with the ^1^H channel (repetition/echo time = 2100/4.2 ms, voxel size = 1.5 mm isotropic, acquisition time = 3:49 min).

A neuroradiologist with 12 years of experience evaluated the FLAIR and SWI using the common data element guidelines developed for TBI.^29^

### ^23^Na and ^1^H MRI post-processing

As previously reported,^9^ the ^23^Na images were reconstructed offline using non-uniform fast Fourier transform^30^ with an in-house MATLAB script. Sodium concentration maps in the brain were computed using the standard deviation of the background noise (560 pixels from the outer corner of the central slice set to 0 mM) and the vitreous humour within the eyes as internal reference (where the concentration is assumed to be 140 mM,^31^ with no relaxation correction) to calibrate the ^23^Na signal.

Global aTSC values in WM and GM were obtained with linear regression over all brain voxels as described in our prior publication.^9^ Briefly, MPRAGE images were segmented into GM, WM and CSF masks using SPM12 (https://github.com/spm/, UCL, UK), to yield tissue volume fractions at each voxel. The probability masks were then re-gridded to the ^23^Na resolution and convolved with the point spread function (mono-exponential T_2_ decay with 56 ms) to counteract CSF spill-over effects. A regression analysis was then applied to the over-determined system of equations (i.e. the computed aTSC, weighted by the tissue volume fractions per voxel) to solve for the two unknowns: aTSC of global WM and aTSC of global GM, separately.

MPRAGE images from the ^1^H MRI acquisition were processed using the FreeSurfer 6.0.0 recon-all pipeline,^32^ which involves the segmentation of subcortical structures. All volume estimates (in cubic millimetres) were extracted from the statistical output file, *aseg.stats*. For each subject, bilateral ventricular volumes were summed and normalized to their estimated total intracranial volume to control for variations in head size. Normalized ventricular volumes were then used to track possible differences in the CSF signal that could influence the ^23^Na signal within CSF-bordering regions.

### Statistical analysis

Sample sizes were determined as described in our prior publication^9^ and in Supplementary material. Subjects were frequency-matched in age and sex, where patients were only matched to controls of the same sex, and of an age that was within five years of the patients’ age.

We performed descriptive analyses (median, percentile, mean, standard deviation, range) for all collected data. Rates of change were defined as the absolute difference between aTSC, RPQ and BTACT scores at v2 and at v1, divided by the number of days between the two visits. Rates of change were also calculated for RPQ and BTACT scores between v3 and v1.

Non-parametric tests (Table 1) were used in all analyses to avoid assumptions about the distribution pattern of the underlying data. The Mann–Whitney U (MW) test was used to compare aTSC measurements between all patients at v2 and controls, and between GOSE-defined recovered or non-recovered patients at v2 and matched controls. A MW test also compared aTSC at v1 between groups, as well as their aTSC changes between v1 and v2. The matched-pair Wilcoxon signed-rank test (WSRT) was used to examine the following within-subject change among subjects: (i) aTSC between v1 and v2, (ii) symptomatology (RPQ) and cognitive performance (BTACT) between v1 and v2, and (iii) RPQ and BTACT between v1 and v3. Spearman rank correlations were used to assess the associations between (i) aTSC at v2 and RPQ and BTACT at v2, (ii) aTSC at v1 and RPQ and BTACT at each follow-up visit, and (iii) the associations between the rate of change of aTSC between v1 and v2 and that of RPQ and BTACT between v1 and v2 as well as between v1 and v3.

Possible volumetric differences between patients at v1 and controls were tested using the MW test and volumetric within-subject change between visit 1 and visit 2 was examined using WSRT.

All tests were conducted at the two-sided 5% significance level, without correction for multiple comparisons. The hypotheses which are tested by these statistical approaches, are summarized in Table 1.

Please see Supplemental Materials for previous use of data acquired from these cohorts.

## Results

### Subject characteristics and clinical outcome at each visit

After the exclusion of four subjects (Fig. 1), 27 subjects, 22 ± 10 (average ± standard deviation) days from their injury date (range 5–53 days) and 21 age- and sex-matched healthy volunteers were examined at v1.^9^ Patient characteristics, described in detail in our prior publication,^9^ are summarized in Tables 2 and 3. Briefly, a majority had reported loss of consciousness and/or alteration of consciousness between 1 and 29 min, without post-traumatic amnesia. The leading causes of mTBI were falls, pedestrian-object collisions and sport-related impacts. The most frequent self-reported symptoms from the RPQ were headaches and poor concentration. Patients had, on average, a negative BTACT composite *z*-score, indicating cognition scores below those expected in healthy populations. Seventy percent of patients were classified as non-recovered according to the GOSE. Not reported previously were patients’ AIS scores. According to the AIS, 22 injuries were minor, 3 moderate, and 1 serious (1 score was not available).

**Table 2:** Demographic parameters, functional outcome scores and MRI findings from mTBI patients

	mTBI (*n* = 27) Visit 1	mTBI (*n* = 20) Visit 2	mTBI (*n* = 15) Visit 3
Female/Male	20/7	14/6	11/4
Age (years)	36±12 (18–60)	35 ± 12 (18–60)	37 ± 13 (19–61)
Time after injury	22 ± 10 (5–53) days	16 ± 3 (11–20) weeks	60 ± 7 (50–72) weeks
Not Hispanic or Latino			
Asian	1 (4%)	–	–
Black or African American	4 (15%)	4 (20%)	3 (20%)
White	14 (51%)	9 (45%)	6 (40%)
Other (e.g. mixed race, not reported)	2 (7%)	2 (10%)	2 (13%)
Hispanic or Latino			
Asian	–	–	–
Black or African American	1 (4%)	1 (5%)	1 (7%)
White	2 (7%)	1 (5%)	1 (7%)
Other (e.g. mixed race, not reported)	3 (11%)	3 (15%)	2 (13%)
Mode of Injury			
Motor vehicle accident	2 (7%)	–	–
Bike accident	3 (11%)	1 (5%)	–
Pedestrian-related collision^a^	5 (19%)	4 (20%)	–
Sports-related collision	4 (15%)	4 (20%)	24 (27%)
Assault	1 (4%)	1 (5%)	1 (7%)
Fall	8 (30%)	7 (35%)	7 (47%)
Other collision^b^	4 (15%)	3 (15%)	3 (20%)
Glasgow outcome scale—extended (GOSE)			
5 (Lower moderate disability)	1 (4%)	0 (0%)	0 (0%)
6 (Upper moderate disability)	14 (52%)	2 (10%)	1 (7%)
7 (Lower good recovery)	4 (15%)	7 (35%)	8 (53%)
8 (Upper good recovery)	8 (30%)	11 (55%)	6 (40%)
MRI findings			
Diffuse axonal injury	2 (7%)	1 (4%)	Patient dropped out
Haemorrhage	1 (4%)	Patient dropped out	

In parentheses are ranges or percentages.

^a^Includes injuries caused by pedestrian-to-bicycle and pedestrian-to-vehicle collisions.

^b^Includes injuries that involved being struck by or against an object.

**Table 3:** Cognitive performance and clinical outcome of patients

			mTBI (*n* = 27) visit 1	mTBI (*n* = 20) visit 2	Change between visits 1 and 2 (*P*-value)	mTBI (*n* = 15) visit 3	Change between visits 1 and 3 (*P*-value)
**GOSE**	*Recovered/non-recovered*		*8/19*	*11/9*		*9/6*	
**RPQ**	*Total RPQ*		*22* ±*11.5 (0–47)*	*12.3* ±*13.6 (0–46)*	***0.0046***	*11.9* ± *13.6 (0–42)*	***0.0084***
	Three-factor model	Somatic	12.3 ± 6.8 (0–30)	6.3 ± 7.6 (0–28)	**0**.**0044**	7.5 ± 68.6 (0–24)	**0**.**029**
		Emotional	4.6 ± 4.1 (0–15)	2.6 ± 3.7 (0–14)	**0**.**024**	2.6 ± 4.0 (0–14)	0.19
		Cognitive	5.1 ± 3.6 (0–12)	3.2 ± 3.4 (0–9)	**0**.**012**	2.2 ± 2.8 (0–9)	**0**.**0039**
**BTACT**	*Composite z-score*		*−0.35* ± *0.67 (−1.44–0.86)*	*−0.08* ± *0.78 (−1.31–1.47)*	***0.0004***	*0.68* ± *0.68 (−0.14–1.85)*	***0.0005***
	Subtests *z*-score	Word list recall	*−*0.45 ± 1.07 (*−*2.67–2.06)	*−*0.01 ± 0.93 (*−*1.53–2.08)	**0**.**004**	0.51 ± 0.85 (*−*0.9–1.82)	0.13
		Short-delay word list recall	*−*0.44 ± 0.87 (*−*1.75–1.49)	*−*0.14 ± 0.99 (*−*1.77–1.78)	0.099	0.35 ± 1.40 (*−*1.51–3.37)	0.094
		Backward digit	*−*0.16 ± 0.88 (*−*1.39–1.64)	*−*0.09 ± 1.05 (*−*2.15–1.68)	0.44	2.27 ± 1.06 (0.03–3.60)	**0**.**0002**
		Category fluency	0.02 ± 1.2 (*−*1.87–2.92)	*−*0.15 ± 1.05 (*−*2.07–1.95)	0.49	0.29 ± 1.14 (*−*1.73–2.12)	0.52
		Number series	*−*0.63 ± 1.44 (*−*3.74–1.36)	*−*0.09 ± 1.11 (*−*2.26–1.65)	**0**.**0033**	0.65 ± 0.85 (*−*0.93–1.65)	**0**.**0005**
		Backward counting	*−*0.5 ± 1.01 (*−*2.44–1.95)	*−*0.01 ± 1.70 (*−*2.86–4.3)	**0**.**027**	0.01 ± 1.52 (*−*2.27–2.77)	0.19

At visit 1, 30% of the patients have recovered from their injury (GOSE = 8), while at visit 3, nine patients are still not fully recovered (GOSE < 8). Total RPQ and composite BTACT scores are presented in italics. Note the reduction of post-concussion symptom severity (i.e. reduced RPQ scores) and the improvement of cognitive scores over time (i.e. increased BTACT *z*-scores). The corresponding *P*-values were calculated with matched-pair Wilcoxon signed-rank tests. *P*-values lower than 0.05 are presented in bold.

The number of patients who completed the imaging and/or the clinical outcome assessments at v2 and v3 are shown in Fig. 1. Twenty-six percent dropped out of the entire study before v2, and a further eleven percent dropped out of the MRI part only. A total of 44% had dropped out by v3. The demographics, GOSE scores and MRI findings of the patients who returned at v2 and v3 are compiled in Table 2. There were no differences in age and sex between the subgroups. Only two patients had MRI findings at v1, which, for the one patient who returned for their follow-up exam, remained unchanged at v2. At v2, the main modes of injury were falls (35%), pedestrian (20%) and sports-related (20%) collisions; and at v3 they were falls (47%), sports-related (27%) and other (20%) collisions (data not shown). Scanning and outcome testing at v2 took place at 16 ± 3 (range 11–20) weeks from injury, and outcome testing at v3 took place at 60 ± 7 (range 50–72) weeks from injury.

The descriptive statistics on the GOSE-based recovery status, RPQ and BTACT at v2 and v3, are given in Table 3. Forty-five percent and sixty percent of patients were classified as non-recovered according to the GOSE at v2 and v3, respectively. There was no difference in terms of recovery status at v1 between patients who returned for their follow-up visit and those who did not return (Supplementary Fig. 1). The most frequent RPQ symptoms at v2 were poor memory (50% of patients, *n* = 10), feeling frustrated, poor concentration (both 45%, *n* = 9), fatigue and headache (both 40%, *n* = 8). Those at v3 were light sensitivity and sleep disturbances (40% of patients, *n* = 6), followed by headache, feeling frustrated, fatigue, poor concentration, poor memory and blurred vision (each 33%, *n* = 5). At v2, the average BTACT composite *z*-score was 0, indicating average cognition scores equal to those expected in healthy populations. At v3, the average BTACT composite *z*-score was 0.7.

Normalized ventricular volumes amongst controls (0.0099 ± 0.0045) did not differ from normalized ventricular volumes amongst patients (0.0096 ± 0.0056) (MW, *P* = 0.50). Normalized ventricular volumes also revealed no changes between v1 and v2 (WSRT, *P* = 0.98).

### Longitudinal changes in clinical outcome (H1)

Serial changes in outcome assessments were calculated using data only from patients who contributed serial data. As shown in Table 3, in comparison with v1, there was a statistically significant difference in total RPQ and composite BTACT *z*-scores at v2 and v3. Specifically, RPQ scores decreased (denoting fewer symptoms and/or symptom burden) while BTACT *z*-scores increased (indicating better performance on cognitive testing). For the RPQ, statistically significant changes were observed across all domains (somatic, emotional and cognitive), and for the BTACT, statistically significant improvements were observed in four out of six subtests. Of those not recovered according to the GOSE at v1, seven were recovered at v2, and eight were recovered by v3.

### Longitudinal changes in aTSC (H2); aTSC at v2 in patients and controls (H3)

**Figure 2:**
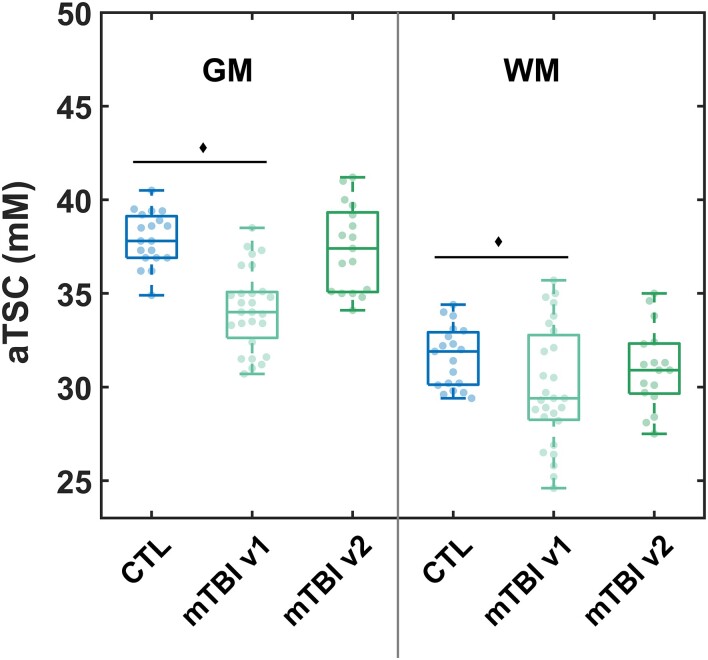
**aTSC in global grey and white matters derived from linear regression.** Boxplots of the sodium distributions in controls and patients at visit 1 (v1, *n* = 27)^9^ and patients at visit 2 (v2, *n* = 17). Grey matter aTSC was decreased in patients at v1 compared to controls (Mann–Whitney U-test, *P* < 0.001),^9^ but not when comparing patients at v2 to controls (*P* = 0.48). Similarly, white matter aTSC was decreased in patients at v1 compared to controls (Mann–Whitney U-test, *P =* 0.042),^9^ but not at v2 (*P* = 0.39).

Boxplots of the patients’ GM and WM aTSC distributions at v1^9^ and at v2 (this study), next to the respective aTSC in controls,^9^ are shown in Fig. 2. The descriptive statistics from which these distributions were derived, are given in Supplementary Table 1. As previously reported, both GM and WM aTSC were lower in mTBI compared to controls at v1 (*P* < 0.001 with Cohen’s *d* = −2.00 and *P* = 0.042 with Cohen’s *d* = −0.60, respectively).^9^ Between v1 and v2, however, there was a statistically significant increase in subject GM aTSC at a rate of 0.04 ± 0.04 mM/day (*P* = 0.0004, Table 4). Subject WM aTSC also increased with time, but without statistical significance (*P* = 0.1). Nevertheless, subject GM and WM aTSC at v2 were no longer reduced in comparison to controls’ (GM *P* = 0.48 and WM *P* = 0.39; Fig. 2 and Table 4). The Cohen’s *d* for the aTSC differences between patients at v2 and controls were −0.27 and −0.31 for GM and WM, respectively.

**Table 4:** Descriptive statistics of the within-subject difference and rate of change in aTSC among patients

aTSC (mM) in mTBI	Difference (v2−v1)				Change rate (v2−v1/Δtime)				WSRT
	Mean	SD	Median	IQR	Mean	SD	Median	IQR	*P*-value
GM	3.6	3.0	2.8	4.2	0.04	0.04	0.03	0.05	0.0004
WM	1.1	2.3	0.5	3.5	0.01	0.03	0.006	0.04	0.10

While the difference depicts aTSC visit 2−aTSC visit 1, the change rate divides the difference by the time between the two visits and is given in mM per day. GM values increased significantly with time for patients according to the matched-pair WSRT.

### Cross-sectional correlations between aTSC and clinical outcome at v2 (H4)

There were no statistically significant correlations between subject aTSC at v2 and RPQ scores, nor between subject aTSC at v2 and BTACT *z*-scores.

The comparisons between controls and recovered patients, and between controls and non-recovered patients in terms of aTSC at v2, also showed no statistically significant differences.

### Prediction of clinical outcome (H5)

**Figure 3:**
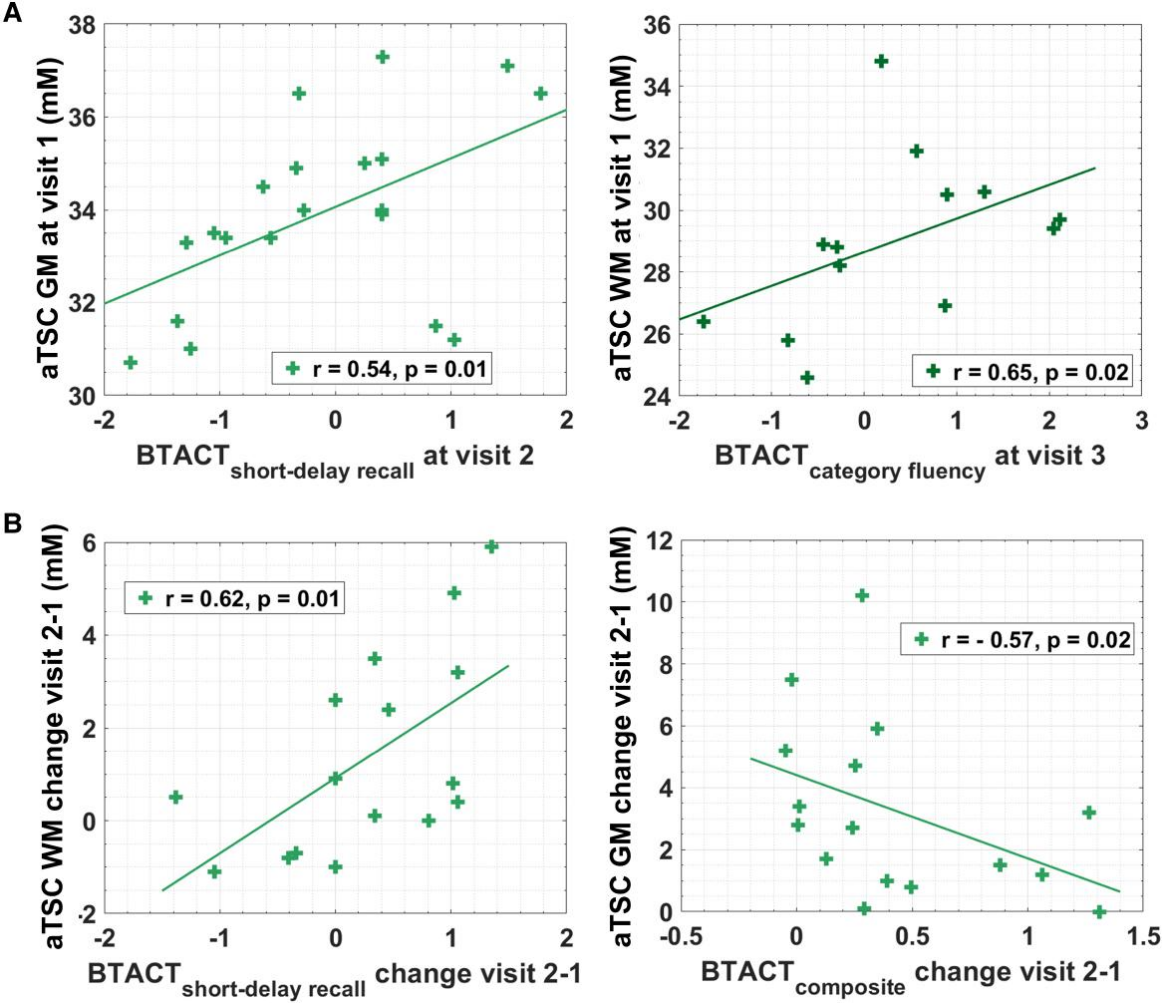
**Prediction of clinical outcome and correlations between changes in aTSC and changes in clinical outcome.** (**A**) Spearman correlations (*r*) and *P*-values are presented for the association of aTSC at visit 1 with BTACT subtest *z*-scores at follow-up visits (*n* = 20 at visit 2 and *n* = 15 at visit 3). No correlations were found between either grey or white matter apparent total sodium concentration and Rivermead post-concussion symptoms questionnaire, a measure of the presence and severity of post-concussion symptoms. (**B**) Spearman correlations and *P*-values are presented for the association of change of aTSC between visit 2 and visit 1 with the corresponding change of BTACT scores (*n* = 16). *P*-values for Spearman’s rho were computed using the exact permutation distributions.

No correlations were found between aTSC at v1 and subject RPQ scores at the two follow-ups. Conversely, GM and WM aTSC at v1 correlated with future BTACT subtests scores (Fig. 3A): GM aTSC at v1 correlated positively with the short-delay recall test score at v2 (*r* = 0.54, *P* = 0.01) and WM aTSC at v1 correlated positively with the category fluency test score at v3 (*r* = 0.65, *P* = 0.02).

The patient cohort at v1 was dichotomized based on the GOSE scores at v2 into future recovered and future non-recovered, and each group’s v1 aTSC was compared to that of matched controls. Statistically lower GM aTSC were found for both non-recovered and recovered group compared to controls (non-recovered versus control: GM *P* < 0.0001, WM *P* = 0.085; recovered versus control: GM *P* < 0.0001, WM *P* = 0.05).

### Correlations between rates of change in aTSC and changes in clinical outcome (H6)

Changes in RPQ scores did not show any statistically significant correlations with changes in aTSC. As shown in Fig. 3B, the magnitude of increases in BTACT short-delay recall test between v2 and v1 correlated with increases in WM aTSC between v2 and v1 (*r* = 0.62, *P* = 0.01); while increases in BTACT composite score between v2 and v1 correlated negatively with GM aTSC change (*r* = –0.57, *P* = 0.01).

The aTSC rates of change in patients (*n* = 8) who did not recover at v1, but recovered at v2, were not statistically different from the rates of change of those (*n* = 7) who did not recover by v2 (GM *P* = 0.2, WM *P* = 0.64).

## Discussion

It is well-established that axonal stretching during a TBI-causing event results in an abnormal ionic exchange across the neuronal plasma membrane.^33,34^ There can be multiple consequences of this ionic imbalance, the most studied of which is axonal cytoskeletal breakdown, which can be identified histopathologically by an amyloid precursor protein stain. It is established, however, that while such axonal injury is a hallmark of TBI, it does not reflect the full scope of cellular damage. Specifically, non-structural, metabolic abnormalities are thought to be more widespread,^33^ and to contribute to clinical disability, especially in mTBI, where cytoskeletal breakdown is not as prominent as in more severe TBI. One example is sodium channelopathy,^35^ which has recently been proposed as an important pathophysiological substrate of neuronal dysfunction. Animal and human histopathology after TBI have documented loss^10^ and long-term structural damage^11^ of Na^+^ channels, as well as of structural disruptions at the nodes of Ranvier.^10^ Such changes are thought to cause action potential-related electrophysiological dysfunction,^12-14^ which in turn may drive patient symptoms or deficits.

The most likely mechanism linking electrophysiological dysfunction to sodium channelopathy is disruption of the cellular electrochemical gradient, which is largely determined by the intra- and extra-cellular concentrations of sodium. Therefore, the ability to detect aTSC with ^23^Na MRI non-invasively, and the potential of discovering clinically relevant aTSC changes in TBI, have motivated our prior work. Based on the novelty of the application, we applied an exploratory approach to uncover all true effects, albeit at the risk of type I errors. The results of all aTSC comparisons between subjects and controls, however, were congruent in demonstrating aTSC decreases throughout the global GM and WM. This effect was best captured with a post-processing technique we adapted from ^1^H MR spectroscopic imaging, in which the mean GM and WM concentrations over many voxels are calculated using linear regression.^36^ The strongest correlations with clinical outcome were also found using the aTSC obtained with this approach. We therefore constrained this follow-up study to global GM and WM aTSC, obtained via linear regression, with the goal of investigating their serial changes, optimizing prediction of future clinical outcome, and to capture the dynamic evolution of symptoms and outcome.

Our first hypothesis (H1) was that subject symptoms and outcomes would exhibit gradual, measurable improvement with time, in accordance with insights from past studies.^3,37-39^ Indeed, there was a statistically significant improvement in almost all outcome scores, supporting this hypothesis. The small sample size of our study prohibits meaningful comparison of the magnitude of improvement to that reported in other studies, but we note the following consistencies between our findings and literature: (i) most commonly reported RPQ symptoms^40,41^; (ii) complete cognitive recovery by 3–12 months^39,42^; (iii) a proportion of GOSE-defined non-recovered patients after 6 months.^3,40,43^

Our second hypothesis (H2) stated that aTSC would concurrently show a serial change towards normalization to control levels, consistent with the expected improvement in patient outcome over time in H1. This hypothesis was also supported: GM aTSC showed a statistically significant increase with time, suggesting recovery of ionic homeostasis. While WM aTSC did not exhibit a statistically significant increase with time, it was no longer significantly reduced compared to controls at v2, due to either insufficient statistical power to observe an increase, or an increase in the coefficient of variation (wider boxplots in Fig. 3A). According to the related hypothesis, H3, we expected smaller effect sizes in the aTSC differences between patients and controls at v2 compared to those at v1. This hypothesis was also supported, due to the normalization trends seen in subject aTSC. We note that there were no longer statistically significant differences between the cohorts at v2.

Within H4 to H6, we investigated the relationships between aTSC and patient outcome. In H4, we postulated that given that aTSC showed cross-sectional correlations with outcome at v1, aTSC at v2 would show correlations with outcome at v2. This hypothesis was not supported in any outcome measure. Our main hypotheses, given their relevance to study aTSC as a biomarker for mTBI recovery, were as follows. Lower aTSC measured at an average of 22 days post-TBI would predict worse outcome at 3 months and 1 year (H5); and serial aTSC changes would correlate with serial changes in patient outcome (H6). Intriguingly, both hypotheses showed similar results in that they were not supported for the RPQ and GOSE but were supported for the BTACT. We note a congruence with the cross-sectional data in terms of the RPQ and BTACT results. Specifically, at v1 we did not find any correlations between aTSC and RPQ, despite having classified the RPQ scores into the additional subsets of the three-factor model and of RPQ-3 and RPQ-13. Hence, the lack of associations between aTSC and RPQ in the serial data (H4–H6) may be interpreted as additional evidence that aTSC changes are independent of symptomatology as measured by RPQ. In contrast, the cross-sectional data showed very strong associations between aTSC and the BTACT, and the results from this study confirm the notion that aTSC is instead reflective of cognitive function. We note, however, that the correlations from the prior report were found with most BTACT subtests and with the BTACT composite score, while here the predictive associations were only found with one subtest at each visit; and there were no cross-sectional correlations at v2. In terms of associations between aTSC and recovery status based on GOSE, while these existed in the previous report, none were found in the serial data.

The above findings cannot be compared to similar studies, because to the best of our knowledge, this is the first serial ^23^Na MRI studies in TBI. There is also a paucity of serial ^23^Na MRI studies in other neurological disorders.^44^ However, the majority of cross-sectional studies which compared aTSC to clinical outcome metrics have found correlations between higher aTSC and worsened outcome.^44^ We previously proposed that the GM and WM aTSC decreases at v1 could relate to cellular swelling and intracellular sodium influx. Although counterintuitive, a small increase in the intracellular volume can result in decreased aTSC, even if the intracellular sodium concentration increases, and potentially even with increasing total tissue sodium content, depending upon concomitant fluid shift.^9^ The increase in aTSC at v2 reported here might therefore reflect normalization of ionic balance and cell volume, but demands more dedicated investigation to better elucidate the nature of this relationship.

### Limitations

The COVID-19 pandemic impacted the final portion of our v1 data collection, and particularly hindered data collection during scheduled follow-up in our longitudinal, prospective study. Alongside the relatively high attrition rates common in mTBI imaging research, this resulted in modest patient sample sizes at v2 and v3. Given also that most patients recover over time, the statistical power to detect associations between aTSC and different recovery trajectories was further negatively impacted. To address this, future studies should plan for a larger proportion of non-recovered individuals at the chronic phase, by recruiting (i) more patients; (ii) higher proportion of patients with CT or MRI findings; or (iii) patients with moderate TBI; as all these groups are likely to have negative outcomes that resolve slower. We note that an additional impetus for studying cohorts (ii) and (iii) is that the presence of MRI-visible injury will provide an opportunity to test the hypothesis that incorporating knowledge of focal injury sites will result in stronger relationships between aTSC and clinical outcome. Indeed, previous work has shown that cognitive deficits depend on lesion site,^45,46^ which is noteworthy in light of our main conclusion, i.e. aTSC is linked exclusively to cognitive outcomes. Studies in more injured cohorts, however, should account for possible atrophy, which can affect aTSC measurements. Specifically, a CSF spill-over effect could result in higher aTSC in CSF-adjacent regions. We cannot exclude the presence of such effect in the current study, but a lack of CSF changes between v1 and v2, along with a lack of differences between regions adjacent and not adjacent to the CSF (Supplementary Tables 2 and 3) support the assertion that the results of the global analysis are independent of any CSF effects.

Another consequence of the COVID-19 pandemic was that scanning intervals were disrupted for periods of several months due to institutional restrictions. It is therefore possible that some patients with disrupted follow-up differed significantly from those who were successfully followed, engendering potential biases if, for example, patients suffering from PCS were more likely to complete their scanning and clinical follow-up regimes compared to asymptomatic patients. Although we did not see a difference in the proportion of asymptomatic patients and patients with PCS between v1 and the follow-up visits based on the GOSE, this could be caused by the relatively small number of participants.

## Conclusion

GM and WM aTSC in mTBI, previously shown to be diffusely decreased compared to controls at v1, were no longer reduced by v2. Coupled with the statistically significant increase in GM aTSC, these findings suggest normalization of the sodium ionic equilibrium. These changes were accompanied by marked improvement in outcome in terms of symptom burden and performance on cognitive testing. Our main hypothesis (i.e. v1 aTSC predicts future outcome) was partially supported, in that aTSC predicted BTACT, but not RPQ scores nor future GOSE status, and that the BTACT findings were only in single subtests at each time point. We note, however, that our prior study also reports aTSC correlations only with the BTACT (and not with the RPQ), lending further support to aTSC as a predictor of cognitive function rather than symptomatology.

## Supplementary Material

fcae229_Supplementary_Data

## Data Availability

The data that support the findings of this study are available from the corresponding author, upon reasonable request. MATLAB script used for the linear regression analysis is available on zenodo (10.5281/zenodo.10996401).

## Supplementary material

Supplementary material is available at *Brain Communications* online. Post-processing code is publicly available with zenodo: 10.5281/zenodo.10996401.
