# Supplementary material for: Longitudinal changes in sodium concentration and in clinical outcome in mild traumatic brain injury
Source: Brain Commun. 2024 Jul 11;6(4):fcae229. doi: 10.1093/braincomms/fcae229 (PMC11258572; doi:10.1093/braincomms/fcae229)
Supplement: fcae229_Supplementary_Data [file fcae229_supplementary_data.docx]

## Supplementary Material

### Inclusion criteria for patients and healthy subjects

The inclusion criteria for mTBI patients in this study were: (*i*) age 18–65 years at start of the study, (*ii*) a diagnosis of mTBI according to the American Congress of Rehabilitation Medicine (1), (*iii*) less than two months from their injury date, (*iv*) no diagnosis of mTBI within the past two years, (*v*) no history of moderate or severe TBI or other neurological disease, (*vi*) no history of disqualifying neurological or psychiatric conditions, or substance abuse, and *(vii)* no MRI contraindications.

Inclusion criteria for healthy controls were an absence of disqualifying neurological conditions, and no MRI contraindications. Exclusion criteria for controls were prior medical diagnoses of mTBI within the past two years and moderate TBI.

### Sample size calculation

The sample size of this study was dependent on the sample size calculations presented in our prior publication (2), which set the sample size so that the study would have at least 80% power to detect a cross-sectional aTSC difference between patients and controls of 20%. For the serial aspect, 35 subjects in each subject group were deemed enough to account for data loss due to technical issues (e.g., excessive motion) and up to 20% attrition. With data from at least 25 subjects per group, the study would have at least 80% power to detect correlations of magnitude 0.316 (R^2^ ≥ 10%) using data from all subjects and magnitude 0.4 among patients. Unfortunately, termination of research activities due to COVID-19, impeded our efforts to achieve full recruitment (27 patients and 19 controls entered the study with full datasets *vs*. the planned 35 in each group). Attrition was also higher than expected (44% vs. planned 20% by v3).

### Previous use of data

For correct accounting of effects in meta-analyses and literature reviews, we list which of the data reported herein has overlap with previous publications. At time of publication, results from this patient and control cohort have been reported in the cited cross-sectional ^23^Na study (2), but also in two other unrelated publications (3, 4). The overlap between the current study and the latter two reports is only in the patient outcome data: GOSE, RPQ and BTACT scores.

**
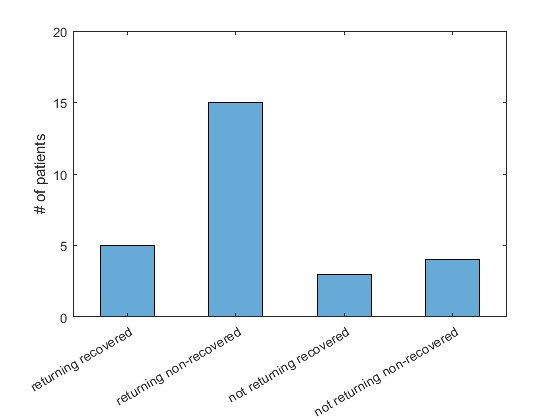
**

**Supplementary Fig. 1: Number of patients that returned to the second visit (returning) or not (not returning) depending on their Glasgow Outcome Scale – Extended outcome of their first visit (recovered: Glasgow Outcome Scale – Extended score = 8 at visit 1; non-recovered: Glasgow Outcome Scale – Extended score < 8 at visit 1).** Patients returned to their second visit independently of their recovery status at visit 1 based on Mann-Whitney U test comparing returning (n = 20) and not returning patient group (n = 7) (*p* = 0.40).

**Supplementary Table 1: Mean, SD, CV, median, IQR of grey and white matter of Apparent Total Sodium Concentration (aTSC) in mM from controls and mTBI cohort for each visit.** Cohen’s d and p-values based on Mann-Whitney U tests were calculated between mTBI at visit 1 and controls and between mTBI at visit 2 and controls.

| **aTSC (mM)** | **mTBI** | | | | | **mTBI vs control** | | **Controls** | | | | |
| --- | --- | --- | --- | --- | --- | --- | --- | --- | --- | --- | --- | --- |
| **Visit 1** | Mean | SD | CV | Median | IQR | MW  p-value | Cohen‘s d | Mean | SD | CV | Median | IQR |
| Grey matter | 34.2 | 2.1 | 0.06 | 34.0 | 2.2 | 0.001 | -2.00 | 37.9 | 1.9 | 0.05 | 37.8 | 2.3 |
| White matter | 30.0 | 3.1 | 0.10 | 29.4 | 4.3 | 0.004 | -0.60 | 30.8 | 2.9 | 0.09 | 31.4 | 2.9 |
| **Visit 2** |  |  |  |  |  |  |  |  |  |  |  |  |
| Grey matter | 37.4 | 2.2 | 0.06 | 37.4 | 4.1 | 0.476 | -0.27 | - | - | - | - | - |
| White matter | 31.0 | 2.1 | 0.07 | 30.9 | 2.6 | 0.392 | -0.31 | - | - | - | - | - |

Abbreviations: CV: coefficient of variation; IQR: interquartile range; SD: standard deviation.

### Regional grey and white matter analysis

We extracted masks of the pallidus and putamen (not adjacent to CSF) and cortical GM, as described in our previous report (2). Similarly to visit 1 (2), at visit 2 (current manuscript) we found higher aTSC in cortical GM compared to the pallidus and putamen. The matched-pair Wilcoxon signed-rank test (WSRT) was used to examine within-subject change for all GM regions. No significant difference was observed between the two visits for the regional analysis.

The same was true when we had a look at the rate of change for these three regions (absolute difference of aTSC at visit 2 minus aTSC at visit 1, divided by the number of days between the two visits). All rates of change were not statistically significant. See Supplementary Table 2 below for details.

**Supplementary Table 2:** **Descriptive statistics of the within-subject difference and rate of change in regional grey matter Apparent Total Sodium Concentration (aTSC) among patients.** While the difference depicts aTSC visit 2 – aTSC visit 1, the rate of change is the difference divided by the time between the two visits and is given in mM per day. No significant regional change with time was observed for patients (matched-pair Wilcoxon signed-rank test (WSRT)).

| **aTSC (mM) in mTBI** | **Difference (visit 2-visit 1) [mM]** | | | | **Rate of change ([visit 2-visit 1]/time) [mM/day]** | | | | **WSRT** |
| --- | --- | --- | --- | --- | --- | --- | --- | --- | --- |
|  | *Mean* | *SD* | *Median* | *IQR* | *Mean* | *SD* | *Median* | *IQR* | *p-value* |
| Pallidus | 0.8 | 2.4 | 0.3 | 1.9 | 0.008 | 0.024 | 0.0038 | 0.023 | 0.177 |
| Putamen | 0.5 | 1.8 | 0.5 | 1.8 | 0.006 | 0.021 | 0.0056 | 0.022 | 0.210 |
| Cortical grey matter | 0.3 | 1.8 | -0.1 | 2.0 | 0.004 | 0.023 | -0.0013 | 0.022 | 0.687 |

Abbreviations: IQR: Interquartile range; SD: standard deviation.

We delineated also masks of frontal white matter (FWM) and posterior white matter (PWM) (not adjacent to CSF) and of the body and splenium of the corpus callosum (CC) (which have neighboring CSF), as described in our previous paper (Gerhalter et al. 2021). Global white matter extracted with Freesurfer is also shown for comparison.

Similarly to visit 1 (2), the WM regions more adjacent to CSF show higher values also at visit 2 (current manuscript). The matched-pair Wilcoxon signed-rank test (WSRT) was used to examine within-subject change for the different WM regions. No significant difference was observed for the regional analysis.

The same was true when we had a look at the rate of change for these three regions. Rate of changes of the two CC regions were either negative or positive. Given the high SD and IQR and the absolute small differences between visit 1 and visit 2, the changes remain insignificant (similarly to the regional GM analysis above).

**Supplementary Table 3**: **Descriptive statistics of the within-subject difference and rate of change in regional white matter Apparent Total Sodium Concentration (aTSC) among patients.** While the difference depicts aTSC visit 2 – aTSC visit 1, the rate of change is the difference divided by the time between the two visits and is given in mM per day. No significant regional change with time was observed for patients (matched-pair Wilcoxon signed-rank test (WSRT)).

| **aTSC (mM) in mTBI** | **Difference (visit 2-visit 1) [mM]** | | | | **Rate of change ([visit 2-visit 1]/time) [mM/day]** | | | | **WSRT** |
| --- | --- | --- | --- | --- | --- | --- | --- | --- | --- |
|  | *Mean* | *SD* | *Median* | *IQR* | *Mean* | *SD* | *Median* | *IQR* | *p-value* |
| CC body | -0.6 | 4.9 | -0.4 | 5.6 | -0.005 | 0.050 | -0.005 | 0.056 | 0.586 |
| Frontal white matter | 0.6 | 1.6 | 0.4 | 2.5 | 0.007 | 0.020 | 0.005 | 0.025 | 0.193 |
| Posterior white matter | 0.4 | 2.1 | -0.1 | 1.9 | 0.005 | 0.028 | -0.001 | 0.022 | 0.381 |
| CC splenium | 0.1 | 5.3 | -0.7 | 4.0 | 0.002 | 0.069 | -0.007 | 0.047 | 0.794 |
| Global white matter | 0.7 | 2.3 | 0.4 | 1.9 | 0.007 | 0.029 | 0.004 | 0.020 | 0.210 |

Abbreviations: CC: corpus collosum; IQR: Interquartile range; SD: standard deviation.

# References

1. Kay T, Harrington DE, Adams R: American Congress of Rehabilitation Medicine, Head Injury Interdisciplinary Special Interest Group. Definition of mild traumatic brain injury. *J Head Trauma Rehabil* 1993; 8:86–87.

2. Gerhalter T, Chen AM, Dehkharghani S, et al.: Global decrease in brain sodium concentration after mild traumatic brain injury. *Brain Commun* 2021; 3.

3. Gerhalter T, Cloos M, Chen AM, et al.: T1 and T2 quantification using magnetic resonance fingerprinting in mild traumatic brain injury. *Eur Radiol* 2021.

4. Chen AM, Gerhalter T, Dehkharghani S, et al.: Replicability of proton MR spectroscopic imaging findings in mild traumatic brain injury: Implications for clinical applications. *Neuroimage Clin* 2023; 37:103325.
